# Adapting the Tobacco Pack Surveillance System Protocol to Assess Electronic Cigarette Packaging: Protocol for a Content Analysis

**DOI:** 10.2196/89325

**Published:** 2026-07-02

**Authors:** Reiley Hartmuller, Jennifer L Brown, Hannah Elise Barker, Qinghua Nian, Kevin Welding, Joanna E Cohen, Katherine Clegg Smith

**Affiliations:** 1 Johns Hopkins Bloomberg School of Public Health Department of Health, Behavior and Society Institute for Global Tobacco Control Baltimore, MD United States

**Keywords:** electronic nicotine delivery devices, packaging and labeling, global health, low- or middle-income country, health warning label, electronic cigarettes, electronic cigarette marketing, tobacco and nicotine products, electronic cigarette packaging

## Abstract

**Background:**

In 2021, a total of 82 million people used electronic cigarettes (e-cigarettes) globally. E-cigarette regulations around the globe vary widely from the product being banned in some jurisdictions to being completely unregulated in others. The Tobacco Pack Surveillance System (TPackSS) was initiated in 2012 to monitor tobacco packs available in 14 low- and middle-income countries with the greatest number of people who smoke. The aim of TPackSS is to assess compliance with country-specific tobacco packaging and labeling requirements and identify marketing features and appeals used on tobacco packaging.

**Objective:**

The objective of this study was to adapt and expand the previous TPackSS protocol to also include disposable e-cigarette devices and their accompanying products: e-cigarette liquids and pods or cartridges.

**Methods:**

E-cigarettes were added to TPackSS data collection in Indonesia in 2022 and in China in 2023. Collection took place in Jakarta, Medan, and Surabaya in Indonesia and Shanghai, Beijing, Chongqing, Guangzhou, Kunming, and Shenzhen in China. A total of 15 neighborhoods per city in Indonesia and 12 per city in China were visited. The TPackSS protocol developed for tobacco products in 2012 to 2013 was used as the foundation for the adapted protocol for e-cigarettes. The adaptation of the original TPackSS protocol followed an iterative process involving extensive discussions among the TPackSS study team, comprising researchers with expertise in tobacco control and 13 years of experience in tobacco product packaging surveillance. The study adapted the sampling frame and sampling strategy, pack collection procedures, photography guide, shipment and translation procedures, and codebook to account for the unique country contexts for e-cigarettes (eg, regulations, market for e-cigarettes, and retailer landscape) and the heterogeneity of e-cigarette packaging observed. These adaptations were based on the review of peer-reviewed literature, white papers, Euromonitor data, and consultation with researchers with expertise in e-cigarette marketing and with in-country public health professionals. Using Indonesia as a pilot country, the protocol was evaluated and further refined post implementation for use in China.

**Results:**

The TPackSS study began in 2012 and was funded by Bloomberg Philanthropies. Data collection took place from September to October 2022 in Indonesia and April to May 2023 in China. Across Indonesia and China, 968 unique e-cigarette products were collected. TPackSS data collection, coding procedures, and study findings are publicly available for use on the TPackSS website.

**Conclusions:**

The protocols presented here, which were used in 2 countries with contrasting e-cigarette markets and regulatory requirements, can be adapted for use in other countries. Findings from use of this adapted protocol can inform policy by providing insights into the design features and marketing appeals of e-cigarette products available on the market, as well as compliance with health warning label requirements where applicable.

## Introduction

An estimated 82 million people globally used electronic cigarettes (e-cigarettes) in 2021 [1]. In many countries, including Indonesia and China, rates of e-cigarette use among youth exceed those of adults [2]. Many countries do not regulate flavors, nicotine strength, or liquid volumes, nor do they require graphic health warning labels (HWLs) on e-cigarette products [3-6].

### E-Cigarette Packaging as Advertising

Tobacco and nicotine product packaging serves as communication real estate for both tobacco company marketing and health communication through required government warnings [7,8]. E-cigarette packages can be designed to resemble other objects (eg, Tic Tac container and technology devices such as mobile phones) [9-11]. They also feature a wide variety of marketing appeals such as themes of fantasy or violence, graphic elements such as cartoon characters, and heavily stylized brand names [10]. Among youth, there is an increased interest in trying fully branded e-cigarette packaging when compared to e-cigarette products with plain packaging [12-14]. E-cigarette package attractiveness is also enhanced by a variety of flavors, vibrant colors, and other branding that targets specific subgroups [12,15].

In addition to company branding (names, colors, textual elements, imagery, shapes, and sizes), e-cigarette packaging can also include HWLs, nicotine concentration, and ingredient lists. HWLs on e-cigarette packaging are associated with reduced e-cigarette use initiation and higher intentions to quit vaping among adults and youth [15-19].

### Study Overview

The Tobacco Pack Surveillance System (TPackSS) study was initiated in 2012 with the goal to collect and analyze tobacco products (cigarettes, kreteks, bidis, and straw cigarettes) and their packaging in low- and middle-income countries with the greatest number of people who smoke. Data collection started in 2013 [20]. Roll your own and smokeless tobacco were added to tobacco product collection in 2015 and 2016, respectively, and heated tobacco products and nicotine pouches were added to the types of products to be collected in 2021 and 2022, respectively. The data collection protocol aims to collect 1 of every unique package available for sale in each country. The data generated from TPackSS data collection can inform policies that reduce appeal and attractiveness of tobacco product packaging and more effective HWL implementation and enforcement.

Beginning in 2022, e-cigarettes were added to the TPackSS data collection protocol. In the context of this paper, the term “e-cigarettes” refers to disposable e-cigarette devices and/or their accompanying products: e-cigarette liquids and pods or cartridges. It also can be used to refer to the packaging of these products when discussing them together as a whole. It does not include reusable e-cigarette devices such as tank systems or e-cigarette accessories and parts. In the work described here, e-cigarettes are not included in our definition of tobacco products.

E-cigarettes were an important product type to add to surveillance as the market has grown significantly since their debut in 2007 [21]. As of 2025, a total of 12 million youth aged 13 to 15 years across 110 countries reported current use of e-cigarettes [22]. Surveillance is critical in a market where regulations vary across the globe, including many jurisdictions with no or partial regulations [22]. The TPackSS protocol needed to be adapted for e-cigarette products because the retailer landscape often differs between e-cigarette and tobacco vendors (eg, e-cigarettes are commonly sold at vape shops). In addition, they are often shaped differently than tobacco products, can be more heterogeneous, and consist of different components (eg, liquids compared to sticks). Indonesia was the first country in which e-cigarettes were collected as part of the TPackSS study in 2022. Since then, e-cigarettes were also collected in China in 2023, with other country collections planned.

This paper presents an adaptation of the existing TPackSS protocol [20], originally developed for tobacco products, to optimize it for collection of e-cigarettes in parallel with tobacco products and the process for adaptation, including implementation evaluation and protocol refinements. We describe the specific protocol changes made based on product and market differences unique to e-cigarettes and reflect on our experience implementing and refining the adapted protocol in Indonesia and China. Through these case studies, we highlight key methodological considerations for conducting similar research in jurisdictions with varied e-cigarette landscapes and illustrate how these considerations may be incorporated into the protocol.

## Methods

### Overview

The original TPackSS protocol was adapted for e-cigarettes using an iterative process to enhance validity and to ensure revisions aligned with the study objectives while maintaining methodological rigor. Initial adaptations were made based on our knowledge of the e-cigarette landscape, country-specific regulations, and e-cigarette products and marketing, informed by the peer-reviewed literature, white papers, Euromonitor data, and consultation with experts in e-cigarette marketing research and in-country public health professionals. All adaptations to the protocol were extensively discussed and agreed upon by the TPackSS study team, comprising researchers with expertise in tobacco advertising, tobacco package HWLs, and packaging design, who have conducted research on tobacco packaging for 13 years. Using Indonesia as a pilot country, implementation of each phase of the adapted protocol was monitored, and the research team held regular debriefings to identify challenges, assess adherence to the protocol, and address any needed clarifications. Reflections made during implementation of the protocol in Indonesia also informed further adaptations to the China protocol. This iterative process of revisions, observation, group discussion and reflection, and refinement enhanced feasibility and data quality while preserving the study’s core objectives.

The original TPackSS protocol included a comprehensive overview of each phase of the research from data collection to dissemination of findings [20]. Here, we present how each step was adapted for e-cigarettes and the factors considered when making adaptations. Descriptions of how the protocol differed in Indonesia and China provide examples of how the protocol can be customized to reflect jurisdiction-specific factors while still adhering to the key steps of the protocol. Table 1 provides a summary of the key differences in the original TPackSS protocol and the protocol developed for e-cigarettes.

**Table 1:** Similarities and differences between the original tobacco product protocol and electronic cigarette (e-cigarette) protocol.

Steps or procedures included in the protocol	Original tobacco product protocol	E-Cigarette protocol
Products collected	Cigarettes, kreteks, bidis, straw cigarettes, roll your own, smokeless, and heated tobacco product sticks	Disposable e-cigarettes, pods or cartridges, and e-cigarette liquids (must contain nicotine)
Vendor types	Various tobacco vendor types based on country context	Various tobacco vendor types based on country context and vape stores
Selecting venues for product purchases	Walking protocol	Preselected vape stores in Indonesia and walking protocol in China
Photography guide	Standard photography guide	In addition to the standard photography guide, steps were added to account for extra packaging layers and variety of e-cigarette product packaging
Shipment and disposal	Shipped product and packaging back to office in the United States	Shipped empty packaging back to office in the United States
Translation	Used professional translation services	Used Chinese-speaking coders (China) or Google Translate (Indonesia)
Codebook	Separate intake, HWL^a^ compliance, and feature and appeals codebooks	Combined intake, HWL compliance (when applicable), and features and appeals into a single codebook and added a nicotine-related section
Features highlighted on the TPackSS^b^ website	HWL noncompliance, flavor appeal, brand family, product type, country, city, collection date, price, less harm appeal, lipstick pack, and stick count	HWL noncompliance, flavor appeal, brand family, product type, country, city, collection date, and price

^a^HWL: health warning label.

^b^TPackSS: Tobacco Pack Surveillance System.

### Study Design and Sampling Framework

#### Defining Inclusion and Exclusion Criteria

Only e-cigarette products that were prepackaged with liquid containing nicotine were purchased, namely, disposable e-cigarette devices and e-cigarette refills (cartridges or pods or premixed liquids).

#### Determining the Geographic Scope

The methods used for selection of cities remained the same as the original protocol, apart from adding 1 city in China [20]. In Indonesia, data were collected in Jakarta, Medan, and Surabaya. In China, data were collected in Shanghai, Beijing, Chongqing, Guangzhou, Kunming, and Shenzhen. Shenzhen was added because of its central role in global e-cigarette production.

In the original protocol, 12 neighborhoods per city, stratified by high, middle, and low socioeconomic status (SES), were selected based on diversity in terms of geographic and residential composition [20]. Neighborhood SES in Indonesia was determined by the number of households living in poverty, as reported in the government’s integrated social welfare database. Neighborhood SES in China was determined by land area, population, and property prices obtained primarily from government websites. To factor in a low concentration of vape stores in Indonesia, 3 additional neighborhoods in each city, 1 per SES strata, were visited, dependent on where vape stores were located; a total of 45 neighborhoods were visited. In China, where vape stores are relatively concentrated, in addition to the original criteria used for selection of neighborhoods, each neighborhood also had to contain a cluster of vape stores; a total of 72 neighborhoods were visited in China.

#### Defining the Sampling Frame

In the original protocol, vendor types were selected based on Euromonitor data on where tobacco products are most frequently sold [20]. To adapt the protocol for e-cigarettes, vape stores were added to the list of vendor types based on Euromonitor data [23]. In China, 2 types of vape stores were identified: (1) branded vape stores that sell primarily 1 name brand (eg, RELX, YOOZ, Snowplus, and MOTI) and (2) nonbranded vape stores that sell many different e-cigarette brands.

#### Developing the Sampling Strategy

In the original protocol, at the first tobacco vendor in each city, every unique tobacco product package for sale was purchased in what was referred to as the index purchase [20]. Similar to the original protocol, every unique e-cigarette package was purchased at the first vape store in each city. The definition of a “unique package” was expanded for e-cigarettes to account for e-cigarette–specific characteristics, defined as any package with at least 1 difference in an exterior feature of the pack, including volume, flavor, nicotine strength, brand name presentation, descriptor, or colors.

Identification and selection of vape stores was intertwined with the selection of neighborhoods. Being less concentrated in Indonesia, the 3 vape stores selected in each city were those with the greatest variety of products, determined based on online descriptions and pictures, with the requirement that they not belong to the same brand chain or owner [24].

After the 3 preselected vape stores were visited in each city in Indonesia, the original walking protocol was followed by data collectors in the remaining 12 neighborhoods in each city. Data collectors purchased unique e-cigarette packages that had not been previously collected from vape stores if they were identified at subsequently visited tobacco vendors.

In China, with a relatively high concentration of vape stores, the original walking protocol was adapted to include identification of branded and nonbranded vape stores. Following the adapted walking protocol, data collectors identified and visited vape stores and tobacco vendors simultaneously.

**Figure 1:**
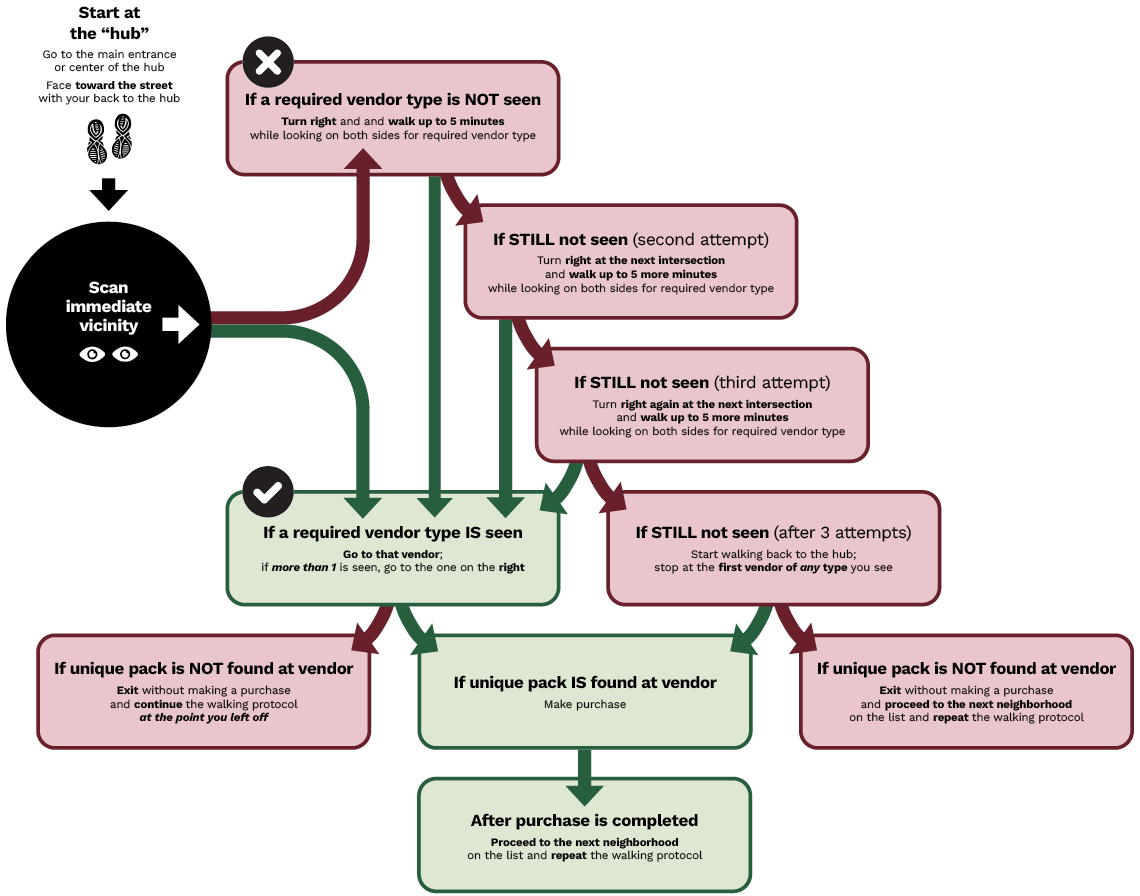
Main walking protocol steps in China. This walking protocol was also followed in Indonesia for tobacco product collection. While not the primary focus, unique electronic cigarette packages were purchased if they were sold at tobacco vendors identified following the protocol.

When following the walking protocol, in each neighborhood, data collectors started at a hub, defined as a central area in the neighborhood. The hub could be a major transit point (eg, bus terminal and train station), historical landmark, tourist attraction, major shopping center, large plaza, square, or major intersection and source of commerce. In the first city visited in China, data collectors started in a neighborhood classified as middle SES and purchased all unique e-cigarette packages from the first vape store they visited. If unique packages were not found at the first vendor visited, data collectors visited up to 2 additional e-cigarette vendors in a given neighborhood until at least 1 unique package was found (Figure 1). At the second and third neighborhood, similar procedures were followed, except either a brand or nonbrand vape store had to be visited depending on the type of vape stores visited previously to ensure both types were visited and variety was achieved. In the remaining 8 neighborhoods, the same procedures for the third neighborhood were used. The entire method was repeated for the remaining 5 cities in China [24].

### Package Collection

The training for data collectors was adapted to contain e-cigarette–specific content and procedures. The specific procedures followed by data collectors to identify unique e-cigarette packages, label e-cigarette packages with unique IDs, create an e-cigarette image archive, document the details of each e-cigarette purchase at the neighborhood level, and create an inventory of e-cigarette packages purchased mirrored the procedures used for tobacco products, as described in the original protocol [20].

### E-Cigarette Photography Guide

The photography guide for e-cigarettes followed the original guide [20], with instructions added to capture additional e-cigarette pack images as needed to account for multiple layers of packaging not commonly found for tobacco products and the greater variety of e-cigarette packaging shapes relative to tobacco products. All layers of e-cigarette packaging were captured, and for e-cigarette packages with unique shapes, all sides that contained descriptors, imagery, or HWLs were captured.

### Shipping and Receiving the Packages

We received updated approval from the US Food and Drug Administration and the US Federal Trade Commission to import and store empty e-cigarette packages for research purposes. E-cigarette products were disposed of prior to shipping; only empty packaging was imported due to safety concerns with shipping batteries and liquid, an issue not encountered with tobacco products. As with tobacco products, packages are stored in our office space in labeled and cataloged boxes. Unlike the intake procedures described previously for tobacco products, intake for e-cigarettes was completed at the same time as coding [20].

### Translation

Since publication of the original protocol, professional translation services were substituted with Google Translate to translate all non-English text on packaging, including tobacco and e-cigarette packaging, into English. Research assistants now use the image translation feature available via the Google Translate smartphone app to record English translations in Microsoft Excel spreadsheets for reference during coding [24]. For the coding of Chinese e-cigarette packages, all team members were able to read Chinese; therefore, translation was not required.

### Creating an E-Cigarette Codebook

The e-cigarette codebook was developed through an iterative process involving incorporation of existing measures, consultation with subject matter experts, in-person review of e-cigarettes collected in Indonesia, and pilot testing followed by refinement. First, we combined our previously separate tobacco product codebooks on intake, warning labels, design features, and marketing appeals into a single codebook to be adapted for e-cigarettes. We then added questions on external packaging, nicotine type and presentation, and cessation claims and expanded flavor and youth appeal response options based on the literature on e-cigarettes and personal observation of e-cigarette packaging. Next, we conducted a literature review to identify existing content analyses of e-cigarette advertising and packaging, identifying 15 relevant peer-reviewed publications [9,25-38]. We conducted informational meetings with research teams that authored 5 of these publications, which used data from Greece, England, and the United States. Prior to the meetings, research teams received a list of proposed codebook constructs (ie, product information, nicotine content, flavors, packaging features, warning labels and messages, marketing messages and images, and advertising tactics). During the meetings, researchers provided feedback on these constructs and responded to targeted questions about their experiences in codebook development, features they would have liked to have coded, coding challenges, and unexpected findings. Detailed notes were taken to document lessons learned, suggestions for improvement, and recommendations for additional codebook constructs. Insights gathered were incorporated into the first full draft of the codebook to enhance its practicality and relevance.

**Figure 2:**
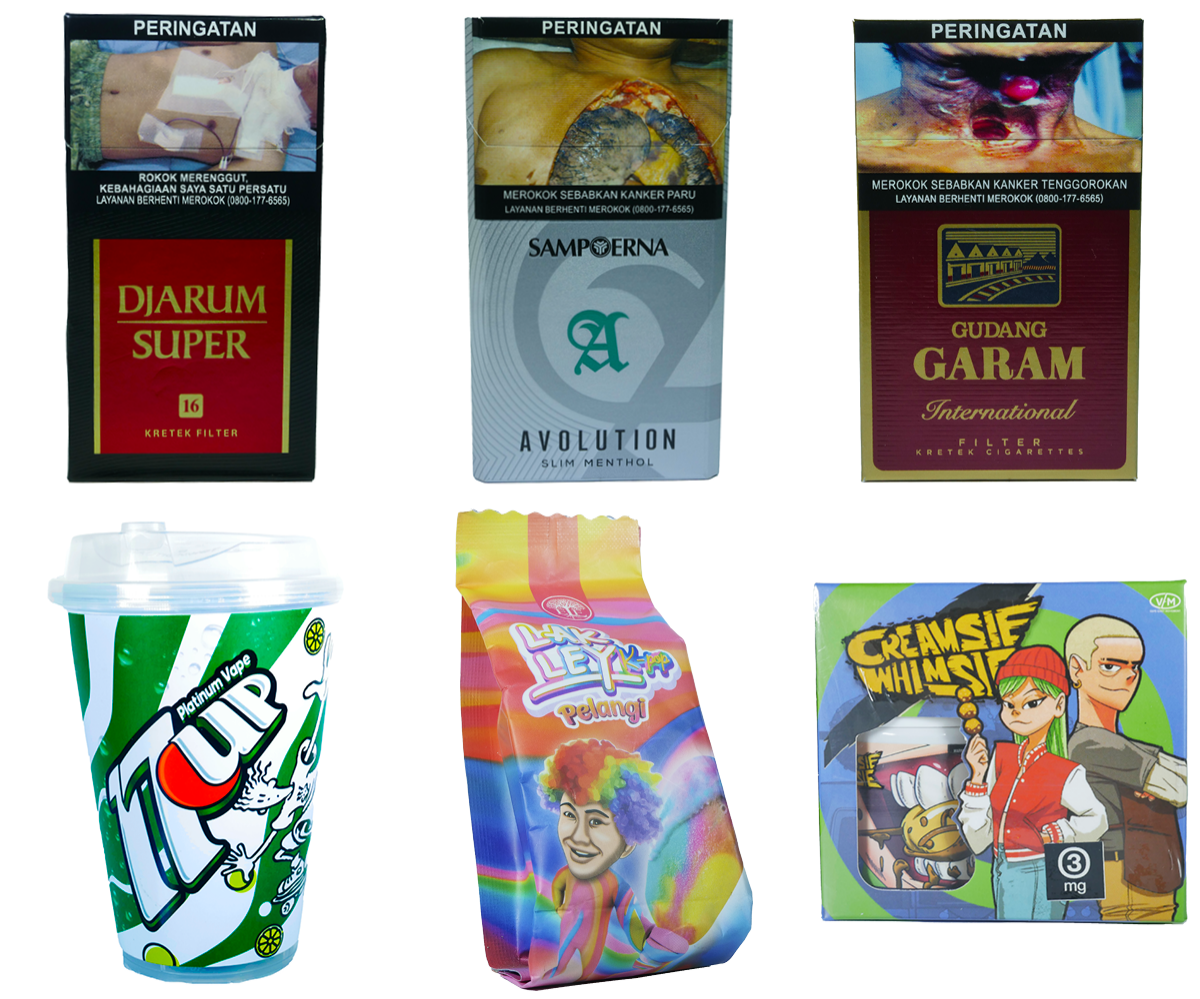
Comparison between cigarette and electronic cigarette (e-cigarette) packages collected in Indonesia, 2022. All e-cigarette images featured are the external packaging of e-cigarette liquids.

To aid codebook development, we also viewed the physical e-cigarette packages collected in Indonesia in person. We found that compared to cigarettes, e-cigarettes from Indonesia came in a greater variety of shapes and displayed unique and varied design elements. The design also seemed more tailored to a younger audience, consistent with the literature on cigarette pack design elements that make cigarettes more attractive to youth and young adults (Figure 2) [39,40]. On the basis of these observations, we added questions and response options to the codebook to capture specific packaging elements likely to appeal to youth (eg, cartoons and fun fonts).

Next, we pilot-tested the codebook with coders and further revised the codebook and accompanying coding guide. A random subsample of 125 packages from Indonesia were used to pilot the first full version of the e-cigarette codebook. Revisions were made for clarity to address package characteristics not previously addressed and to eliminate items that had low interrater reliability. For example, we found the units used to communicate nicotine concentration varied (eg, percentage, “mg,” and “mg/mL”) and added response options accordingly. We also found that “masculine” and “feminine” appeals and brand names could not be determined reliably; these codebook questions were eliminated or edited.

A high level of intercoder agreement was reached for most variables (80%); however, for marketing appeals, there was a trade-off between simplifying the codebook to achieve higher agreement and including appeals that could provide greater context to the marketing being used on the packaging, resulting in lower levels of initial agreement (50%-70%) on some variables.

The codebooks for Indonesia and China e-cigarettes also varied due to different packaging and labeling regulations. At the time of data collection, e-cigarettes were unregulated in Indonesia; therefore, no questions applicable to compliance with existing policies were included in the Indonesia e-cigarette codebook, and only the external packaging layer visible to the consumer at point of sale was coded. Given the existing e-cigarette packaging and labeling policies in China at the time of data collection, the China e-cigarette codebook included related items, and coders were instructed to observe all layers of the packaging and the product. Further revisions were made to the China codebook based on packaging elements specific to Chinese e-cigarettes, additional pilot testing of 10 e-cigarette packages from China, and decisions to simplify the codebook after determining that certain items were not of high priority.

The base codebook structure used in both countries contained 4 sections: product information, nicotine, warning labels, and marketing appeals (Table 2), with some codes only included in the Indonesia or China codebook based on lessons learned and country-specific regulations and context.

**Table 2:** Sections of the codebook and variables used in Indonesia and China (full codebooks are available on the Tobacco Pack Surveillance System (TPackSS) website [24])^a^.

Section	Variables
Basic product information	Duplicate pack status^b^ Purchase date Price Product type External packaging layers Number of puffs as stated on the pack (for disposables)^b^ Number of pods or cartridges^b^ Brand names Manufacturer name^b^ Manufacturer location^b^ Presence of English^c^ Web presence^c^ Insert or onsert
Nicotine	Mention of nicotine Type of nicotine composition Total amount of e-cigarette^d^ liquid Nicotine concentration units Nicotine concentration amount
Health warning and labeling–related items^e^	Presence of text and/or image warnings^c^ HWL^f^ location (for each layer: product, internal packaging, and external packaging)^b^ Observance of HWL through the internal packaging layer^b^ HWL obstruction (for each layer)^b^ HWL in Chinese language (for each layer)^b,g^ HWL text (for each layer)^b,g^ HWL located on bottom of the pack face (for product and external packaging)^b,g^ Branding beneath HWL (for product and external packaging)^b,g^ HWL color (for product and external packaging)^b,g^ HWL text contrast (for product and external packaging)^b,g^ Part of the HWL not present (for product and external packaging)^b,g^ HWL area (for external packaging)^b,g^ Misleading descriptors (for any layer)^b,h^ Health-promoting descriptors (for any layer)^b,h^ Minor protection descriptors (for any layer)^b,h^ Minor protection imagery (for each layer)^b,h^
Features and appeals (coded for on any layer in China and only for the most external visible packaging layer in Indonesia)	Branding information on insert or onsert^b^ Presence of branding information on each packaging layer^b^ Web presence (for each layer)^b^ Presence of company phone number (for each layer)^b^ Marketing claims Claim of cessation aid^c^ Mention of recognition by award or standard^b^ Environmentalism and civic responsibility lexical and imagery appeals^b^ Technology appeal^b^ Taste or sensation terminology Flavor terminology or imagery (for external packaging) Full flavor name^c^ Description of tobacco flavor (for external packaging)^b^ Feminine terminology or imagery^c^ Masculine terminology or imagery^c^ Presence of youth appeal^c^ Specific youth appeals^c^ Youth appeals by text, imagery, and shape^b^ China-specific cultural appeals^b^ Global or luxury or quality appeals^b^ Other notable appeals

^a^Web presence appears in separate sections of the Indonesia (basic product information) and China (features and appeals) codebooks.

^b^Variable appears only in the China codebook.

^c^Variable appears only in the Indonesia codebook.

^d^E-cigarette: electronic cigarette.

^e^All items included in the China codebook are government mandated.

^f^HWL: health warning label.

^g^Repeated for each HWL.

^h^Variables were coded for presence on any layer of the packaging, instead of focusing solely on the HWL.

### Coding

Coding of e-cigarette packages followed the same procedures as previously described for tobacco products [20]. Packages were double-coded independently, and discrepancies were resolved by a third reviewer. Since 2021, we have transitioned to coding all products online using pack images generated using the photography guide rather than in person using the physical packages.

Coders used an extensive coding guide with questions, variable definitions, and package examples as a reference during coding. During a virtual training, coders were introduced to the codebook, coding guide, and coding platform Research Electronic Data Capture (REDCap; Vanderbilt University) and practiced coding packages as a group [41]. As with tobacco products, weekly coding review meetings were held to discuss any unusual packages encountered during coding and resolve coding discrepancies.

For Indonesia, separate qualitative coding of cultural appeals was conducted because the original coders were not all native to Indonesia or closely familiar with its cultural context and, therefore, were not able to adequately assess the meaning of some package elements. Two native Bahasa Indonesian speakers who were raised in Indonesia examined all packages and made an initial assessment of whether text or imagery referred to anything related specifically to Indonesia (eg, a popular character in pop culture, food, or a slang phrase); discrepancies were discussed and resolved by a third party when needed. Next, one of these initial coders reviewed the images of the e-cigarette packages again and wrote a short qualitative description (2-4 sentences) of the cultural appeal. A third study member, who was born in Indonesia, conducted a thematic analysis of the cultural appeals across packages and summarized the results. A fourth study member, who was born in Indonesia, reviewed all packages to determine if cultural appeal overlapped with youth appeal and, when appropriate, categorized and described the culturally specific youth appeal. Two additional Indonesians reviewed these youth appeal categories and descriptions and provided additional insight on whether the appeals were mostly known and liked by younger consumers or if they also appealed to a wider audience. For China, cultural appeals were coded quantitatively as part of the standard codebook because all members of the study team were native to or closely familiar with the Chinese cultural context [24].

## Results

### Packages Collected

The TPackSS study began in 2012 with funding from Bloomberg Philanthropies’ Bloomberg Initiative to Reduce Tobacco Use. Between September and October 2022, we collected 825 unique packages in Indonesia, comprising 782 e-cigarette liquid bottles, 26 disposable e-cigarette devices, and 17 disposable pods or cartridges. Of the 825 unique packages collected in Indonesia, 483 were collected in Jakarta, 203 in Surabaya, and 139 in Medan. Between April and May 2023, we collected 143 unique packages in China, comprising 5 disposable e-cigarette devices and 138 disposable pods or cartridges. No e-cigarette liquid bottles were collected in China. This is likely because the law prohibits the sale of e-cigarette products that allow people to add their own e-liquid to a device [42]. Of the 143 unique packages collected in China, 41 were collected in Shanghai, 31 in Beijing, 6 in Chongqing, 25 in Guangzhou, 15 in Kunming, and 25 in Shenzhen. Data analyses for Indonesia are completed, and secondary analyses are being completed for China. Detailed findings from these collections are being disseminated through separate publications scheduled for 2026 [43].

### Presentation on the TPackSS Website

As with our tobacco product collections, we upload images of the e-cigarette packages to the TPackSS searchable online database [20]. Similar to tobacco products, information about a specific pack is provided along with its photos. Individual e-cigarette packages have the same description categories as tobacco products: brand family, product type, country, city, collection date, and price. Additionally, similar to tobacco products, these packages can be filtered and searched by product type, country, brand family, collection year, and specific features. Some of the current features available for tobacco products (ie, HWL noncompliance and flavor appeal) are applicable to e-cigarettes.

The TPackSS website also features a “Share A Pack” section where users can upload and share photos of tobacco packs from their country. These user submissions contain similar description categories and searchable features to the official uploaded TPackSS collections. This user submission feature has been expanded to encourage uploads of e-cigarette products.

As with previous tobacco product collections, in addition to uploading photos of the packages collected to the project website, we are continuing to create and disseminate country-specific fact sheets. These fact sheets are available for download from the website [24]. The field procedures and codebooks used for these collections and our publications on TPackSS findings are also available via the website.

## Discussion

This protocol provides procedures for systematically purchasing unique e-cigarette products; assessing the products and their packaging for HWL compliance, nicotine information, and design features and marketing appeals; and disseminating the findings. This protocol can be adapted to other countries by taking into consideration the unique policy and market context. The protocol is applicable and adaptable to multiple policy and market contexts. It draws strength from being adapted from the TPackSS project’s years of experience conducting similar work with tobacco products. The protocol has also been informed by the experience of other research teams that have conducted research on e-cigarette packaging.

The adapted protocol was successfully used to obtain or purchase a wide collection of unique e-cigarette packages sold in Indonesia and China. E-cigarette coding resulted in policy-relevant findings that address the study’s main objectives to assess e-cigarette compliance with country-specific packaging and labeling regulations and identify marketing tactics being used on packaging. Findings have been disseminated through fact sheets, peer-reviewed journal publications, academic presentations, and communications with public health professionals in Indonesia and China.

The TPackSS e-cigarette protocol does have some limitations. While the data collection protocol is designed to ideally capture every unique product on the market, it is possible that some unique products are available at vendors we did not visit. In addition, this approach does not allow for the capture of the actual market share of collected products as information on sales is not collected. Products exclusively sold through online retailers or vendors are not captured. For countries where not all coders are culturally tied to the study country, it is possible that specific cultural nuances are missed during coding. Furthermore, coding of appeals such as youth and cultural references involves subjective interpretation. Despite these limitations, this protocol provides a valuable structure for monitoring e-cigarette products and packaging as well as compliance with HWL regulations. Beyond narrative findings, the protocol also ensures the production of visual data that can be incorporated into dissemination materials for diverse stakeholders and aids clear and accessible communication of study findings.

The e-cigarette market is changing rapidly, and continual monitoring is required to inform regulations to protect the public’s health. TPackSS data collection of e-cigarettes will be repeated in the future based on warning labels and packaging requirement changes. The findings from these collections can inform policy by providing insights into the design features and marketing appeals of e-cigarette products available on the market, as well as compliance with HWL requirements where applicable.

## Abbreviations

e-cigarette: electronic cigarette
HWL: health warning label
REDCap: Research Electronic Data Capture
SES: socioeconomic status
TPackSS: Tobacco Pack Surveillance System

## Data Availability

The data supporting this research are available from the authors on reasonable request.
